# Evaluation of laparoscopic knot training in a silicone model

**DOI:** 10.1590/0100-6991e-20233476-en

**Published:** 2023-02-17

**Authors:** LUIZ FELIPE FERNANDES OSORIO, CLARICE FRAGA ESTEVES MACIEL, CARLA BRAGA MANO GALLO, FRANCISCO J. SAMPAIO

**Affiliations:** 1 - Universidade do Estado do Rio de Janeiro, Unidade de Pesquisa Urogenital - Programa de Pós-Graduação em Fisiopatologia e Ciências Cirúrgicas - Rio de Janeiro - RJ - Brasil

**Keywords:** Laparoscopy, Training Courses, Silicones, Surgery, Suture Techniques, Cursos de Capacitação, Modelos Anatômicos, Cirurgia Geral, Suturas

## Abstract

**Introduction:**

in videolaparoscopic surgery, movements are conducted from a twenty times magnified image of an indirect operative field. The video interface used assumes the need for depth perception using two dimensions instead of three.

**Objective:**

to evaluate the effectiveness of training to perform the laparoscopic knot in a silicone model, in 8-hour courses, and to analyze the correlation of learning outcomes with factors such as: sex, age, laterality, previous knowledge in endosuture and medical specialty.

**Material and Methods:**

in this prospective and randomized study, 56 students were evaluated, who took 8-hour courses, with groups of up to ten students. We used: a white box with camera, LCD screen and silicone piece. Four exercises were performed on the silicone mold: right hand, left hand, needle at 45° and back hand.

**Results:**

56 students (mean age = 33.28 years). The female group, n=18, mean age 29.61 years, 17 right-handed and 1 left-handed. The male group, n=38, mean age 34.57 years, 35 right-handers, 1 left-handed and 2 ambidextrous. In both groups, no correlation was observed between the analyzes of well performed knots when correlated with the age or sex of the participants.

**Conclusion:**

laparoscopic knot training, in silicone molds, in 8 h courses, proved to be effective. Factors such as gender, age, laterality, previous knowledge in endosuture and medical specialty do not interfere with the learning results.

## RESUMO

**Introdução:**

na cirurgia videolaparoscópica os movimentos são conduzidos a partir de uma imagem magnificada de vinte vezes de um campo operatório indireto. A interface de vídeo utilizada pressupõe a necessidade de percepção de profundidade utilizando duas dimensões ao invés de três.

**Objetivo:**

avaliar a eficácia do treinamento de execução do nó laparoscópico em modelo de silicone, em cursos de 8h, e analisar a correlação dos resultados de aprendizagem com fatores como: sexo, idade, lateralidade, conhecimentos prévios em endosutura e especialidade médica.

**Material e Métodos:**

Neste estudo, prospectivo e randomizado, foram avaliados 56 alunos, que realizaram cursos de 8h de duração, com turmas de até dez alunos. Foram utilizados: uma caixa branca com câmera, tela de LCD e peça de silicone. No molde de silicone foram executados quatro exercícios: mão direita, mão esquerda, agulha a 45° e back hand.

**Resultados:**

56 alunos (média de idade = 33,28 anos). O grupo feminino, n=18, média de idade 29,61 anos, 17 destras e 1 canhota. O grupo masculino, n=38, média de idade 34,57 anos, 35 destros, 1 canhoto e 2 ambidestros. Em ambos grupos não foram observados nenhuma correlação entre as análises de nós bem executados quando correlacionados com a idade ou sexo dos participantes.

**Conclusão:**

o treinamento de nó laparoscópico, em moldes de silicone, em cursos de 8h de duração, mostrou-se eficaz. Fatores como sexo, idade, lateralidade, conhecimentos prévios em endosutura e especialidade médica, não interferem nos resultados do aprendizado.

## INTRODUCTION

In surgery and other medical specialties, the transfer of skills was performed through a model where the surgeon-in-training played the role of an apprentice. This model, known in the United States and Europe as the William Halsted model, was introduced at Johns Hopkins University in 1889 and served conventional surgery well for over a century^1^.

With the advent of laparoscopic cholecystectomy, there was a rupture in this traditional teaching model. A new condition imposed the need to acquire specific skills, in no way similar to practices in conventional surgery until then, in addition to the need to train surgeons who had already completed their training cycle and were in a teaching position^2^ ^-^ ^4^.

The methods or strategies commonly used to transfer skills in laparoscopic surgery can be divided into two phases: pre-clinical and clinical. The pre-clinical phase comprises the acquisition of theoretical knowledge, training in inanimate models (black boxes, white boxes and simulators) and training in laboratory animals. The clinical phase, the preceptorships.

According to Kopta^5^, for the acquisition of a motor skill, three phases can be individualized.

- Cognitive: This phase involves knowing what to do without necessarily being able to do it. It is about having the theoretical knowledge necessary to take a certain step.
- Integrative: in this phase the student needs to imagine the task and plan all the necessary motor steps for its accomplishment. Repetition improves the flow of movements, so that the execution of the task does not require a cognitive trigger.
- Autonomous: At this stage the performance becomes smooth and automatic. Reaching this stage can signify some level of competence.

In the eighties, Prof. Zoltan Szabo of the MOET Institute, San Francisco, California, USA developed a suturing technique based on a single stitch with another single inverted knot, plus a third knot just to complement. This knot was called square knot, initially developed for microsurgery and performed with the aid of a microscope. In the nineties, with the advent of laparoscopic surgery, Prof. Szabo together with Prof. Alfred Cuschieri adapted this suture training for laparoscopic surgery^6^.

Accurate tissue approximation is essential for the surgical repair of defects and the performance of a safe anastomosis. In addition to a delicate handling of the fabric and careful dissection, tissue approximation must be performed without compromising vascularization, essential for the healing process^6^. 

In open surgery, with three-dimensional vision and with the surgeon’s hands in the visual field, surgical maneuvers become so ingrained and established that knots and sutures are performed semiautomatically.

The advent of laparoscopic surgery has brought with it some challenges that are common to all modalities of laparoscopic surgery and that do not exist in conventional or laparoscopic surgery. The video interface presupposes the need for depth perception using two dimensions instead of three, a limitation corrected with the advent of robotics^7^.

The need to install portals in the abdominal cavity creates the limitation of the fulcrum effect, that is, the need to operate with instruments whose range of motion is limited by a fixed point on the abdominal wall, a limitation that was also attenuated by the instruments used during robotics assistance^7^.

 The consequence of the fulcrum effect causes the surgeon to move his hand in the opposite direction to the tip of the instrument. Tactile feedback is significantly diminished as a result of the separation between the surgeon’s hands and the structures to be operated on. Finally, as a result of the reduced interaction between the surgeon’s hands imposed by the video access route, there is a tendency for surgeons in training to ignore the non-dominant hand, a very common, counterproductive and potentially dangerous occurrence^7^.

This study aims to evaluate the effectiveness of training for doctors, in a suture course, with a workload of 8 hours, in the execution of the laparoscopic knot (square knot) in silicone; analyze whether factors such as sex, age, laterality, knowledge in endosuture, medical specialty and previous experiences interfere in the learning outcomes; assess whether well-executed knots are related to the specialty and evaluate whether well-executed knots depend on the student’s experience with videolaparoscopy; to analyze the correlation of the parameters of execution of knots with the right hand, left hand, needle at 45º and back hand.

## MATERIAL AND METHODS

The training courses were offered to physicians of different specialties, without prerequisites, with guidance for knowledge and practice with the laparoscopic silicone knot, they were given in public and private hospitals, as well as in a study center, free of charge, carried out by a single instructor and evaluator.

There were no conflicts of interest with public or private institutions.

To complete the course, only the informed consent of each student was required for analysis and publication of result data. Ethics Committee does not apply for this type of study.

### Student selection and Data collection

In this prospective, randomized study, we aimed to evaluate the effectiveness of training for physicians (men and women), without prerequisites, with courses taught by a single instructor and evaluator. Six face-to-face courses were held, from July 2020 to July 2021. In the courses we evaluated 56 students, with a workload of 8 hours per course, with small breaks, with groups of up to ten students. 

Before starting the course, the students filled in questionnaires with informed consent and personal data and at the end of the course they answered another questionnaire regarding the effectiveness of the course.

At the end of the course, the instructor individually evaluated the silicone molds of each student.

### Evaluation

For data collection and evaluation of the executed knots, the following questionnaires were applied:

1. Student data

Containing: Name / Age / Gender / laterality / Address / Phone / e-mail / Hospital / Surgical training / Video training / knowledge in video surgery / Specialty / Prior training in endosuture / Difficulties for performing videolaparoscopy surgeries.

2. Quality assessment of the laparoscopic knot

The instructor carried out a careful observation of the stitches on the silicone, evaluating the quantity of: good stitches, loose stitches, undone stitches, tight stitches (torn silicone), stitches not performed and total stitches performed by each participant.

3. Course evaluation (completed by the student at the end of the course)

With positive or negative answers: the use of the course, better handling of the thread, better handling of the needle, improvement in suturing, safety to operate, and indication for other surgeons were questioned. 

Subsequently, all data are tabulated in a spreadsheet for statistical analysis in the GraphPad software.

### Surgical equipment and supplies

**Figure 1:**
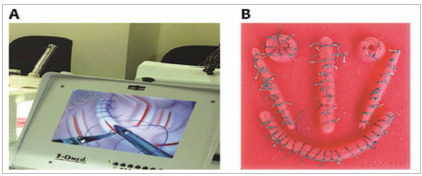
Equipment. A) White box with LCD screen (T3 Series Minimally Invasive Training System, Ohio, USA, www.3-dmed.com/catalog). B) Silicone mold for suture training.

The following equipment were used: a white box with camera and LCD screen to simulate a laparoscopic equipment (one box per student) (Figure 1A), two 12mm trocars, a laparoscopic needle holder, curved Maryland forceps of 5mm, 5mm curved scissors, silicone piece (Figure 1B), 2-0 suture thread with 22mm 1/2 cylindrical needle.

In the silicone mold, the exercise with the right hand was performed on the oblique suture line on the left, and the exercise with the left hand was performed on the oblique suture line on the right. The 45° needle exercise was performed on the midline of the suture and the back hand exercise was performed on the arcuate lower line.

### Suture techniques and knots

In this work, we use the reinforcement of learning feedback using graphic animation.

The student stands up, with relaxed shoulders and arms slightly bent at an angle of 45 to 50°. Movements should be performed with low amplitude for the purpose of more delicate and precise movements.

Regarding the positioning of the trocars for the execution of the suture, it must be such that the camera is in the middle, between the two trocars, positioned laterally so that the instruments pass through the trocars at an angle of 45° to each other.

Initially, the student receives guidance as to the positioning of the suture thread for apprehension and placement inside the black box, using the Maryland forceps, with the hand opposite the hand of the needle holder.

The needle was placed on a surface to facilitate its grasp, due to the lack of depth in the video, which works in two dimensions. The auxiliary forceps must hold the thread 3cm from the needle to position it.

**Figure 2:**
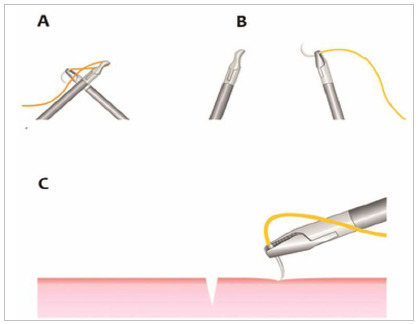
ABC -ABC - Demonstrations of how to adjust the needle in the needle holder.

The needle after grasping can be adjusted in three different ways in the needle holder: by the thread, by the needle or using the surface used in the training, touching the needle tip and opening the needle holder jaw a little for a brief rotation (Figure 2A, 2B, 2C).

**Figure 3:**
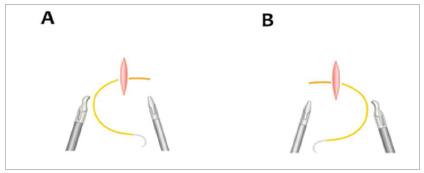
Demonstration of the thread exposure to perform the knot simulating letters. A) letter C and B) letter D.

To make the knot, the thread must pass through the structure and rotate the needle medially, forming with the thread a curvature simulating a letter C, if the suture is being performed with the right hand. If done with the left hand, the needle must be rotated medially, simulating a letter D (Figure 3).

Holding the thread, approximately 3cm away from the needle, with the needle holder, the forceps should be placed on the thread, making a loop with the thread over the forceps for the execution of the first knot. The needle will pass to the other side, being rotated medially, simulating a letter D. Then, repeating the same movement, the second knot is made. Thus, if the exercise was performed with the right hand, a “C D C” is obtained, and if it is performed with the left hand, a “D C D” will be obtained (Figure 3).

### Demonstration of exercises

**Figure 4:**
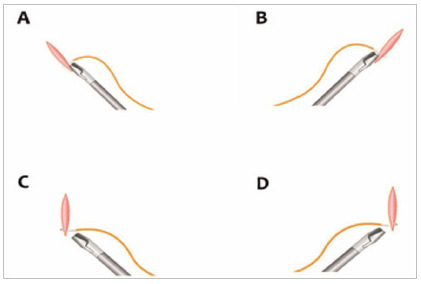
A) Suturing exercise with the right hand and needle at 90° in the needle holder. B) Suturing exercise with the left hand and needle at 90° in the needle holder. C) and D) Suture with both hands, with a needle at 45° in the needle holder.

The first exercise performed was a suture with the right hand. The incision was oblique to the left, the needle holder of the right hand parallel to the suture line and the needle positioned at a 90° angle with the suture line and with the needle holder (Figure 4A).

In the second exercise, the suture line was oblique to the right and the hand to be used was the left. In this case, the needle holder was parallel to the suture line, the needle perpendicular to the needle holder and suture line (Figure 4B).

In the third exercise, the incision was perpendicular to the midline. As the incision is positioned in the middle, the needle holder could be in the right or left hand, but no longer parallel to the suture line. Therefore, for the needle to be perpendicular to the suture line, it should be angled at 45° with the needle holder towards the tip of the instrument. This was done in order to maintain the same distance from the suture edges, aiming at adequate co-option of the suture line edges (Figures 4C and 4D).

**Figure 5:**
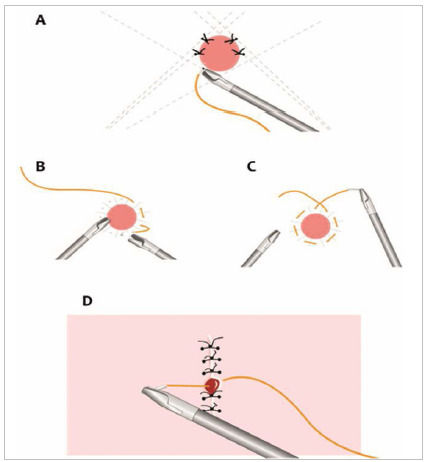
A) Introduction of the back hand stitch from the bottom up. B and C) Continuous suture in a “tobacco bag”. D) Demonstration of the minimum space between two points.

In this exercise, eight stitches were performed, from the outside to the inside, in the north, south, east and west cardinal positions, in addition to intermediate stitches. In the cardinal points, the needle is positioned at 45° in the needle holder. In the intermediates at 90° and in the low points, the suture is performed by holding the needle holder with the inverted hand (back hand), always turning the thread downwards for the execution of the knots (Figure 5A).

In this other exercise, a continuous purse-string suture was performed, from left to right, clockwise and changing hands for each stitch, simulating a tobacco bag, respecting the best hand and needle angle (Figure 5B and 5C).

The distance between two points should be less than the thickness of the instrument, which is half a centimeter (Figure 5D).

### Statistical analysis

The following parameters were analyzed: gender, age, laterality, training with endosuture, specialty, evaluation of well-executed knots related to the specialty, evaluation of good/well-knotted knots and the student’s experience with video, and knot execution parameters with right hand, left hand, 45^o^ needle and back hand.

All parameters were statistically analyzed and graphically described from the average of the values found in each of the analyzed parameters. The Kolmogorov-Smirnov test was performed to ensure normality and the ANOVA test was used to compare the data. Simple linear regression was performed to analyze good/well executed knots, correlating with age, for the groups of participants (male and female). Statistical analysis was performed using the Graphpad Prism software (version 6.01). The p value ≤0.05 was considered statistically significant.

## RESULTS

### Gender, age, laterality, endosuture training and specialty

We analyzed 56 students (doctors) divided into two groups: male group and female group. The mean age was 33.28 years (23-60 years).

Male group: (n=38) mean age 34.57 years (26-60 years), and 90% aged between 26-35.3 years, 35 were right-handed, 1 left-handed and 2 were ambidextrous. 

Women Group: (n=18) mean age 29.61 years (23-47 years), with 90% aged between 23 and 30.9 years, 17 had right-handedness and 1 left-handed.

For the analysis of percentage of laterality, referring to the total of 56 students, 93% with right laterality were observed as a result; 3% left-handed and 4% ambidextrous.

Of the total number of students, 57% had no training with endosuture, 64% had a specialty in general surgery, 12% in urological surgery, 11% in gynecological surgery, 4% in colorectal surgery, 9% had no specialty.

### Evaluation of well-executed knots related to the specialty

The following results were observed for well-knotted knots: an average of 26,0% (19-41%) of good knots when performed with the right hand; 24.0% (22-26%) when performed with the left hand; 21,4% (18-23%) when performed with the needle at 45º and 28.6% (15-33%) when performed with the back hand. No statistical difference was observed when the parameters were correlated with each other (p=1,000).

### Evaluation of good/well executed knots and the student experience with video

Knot performance data were correlated with the number of good/well executed knots, being analyzed in male and female groups, who reported having experience with video, experience with advanced video or no experience. The results were similar for the correlated parameters of experience or not with video, with no statistical difference between the groups (p=1,000).

### Statistical analysis with Linear regression

**Figure 6:**
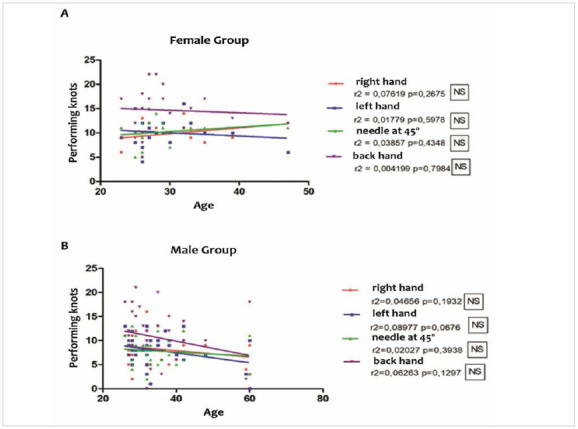
Linear regression graphs - Evaluation of the groups: A) female and B) male, in terms of age and number of good/well performed points.

The parameters for performing well-executed knots with the right hand, left hand, needle at 45º and back hand in the female and male groups were correlated with the age of the students. No significant correlation was observed (Figure 6).

### Course evaluation and learning performance

In the final questionnaire, answered by the students, referring to the evaluation of the course, the result indicated a percentage of 83.3% of positive answers, for general use of the course and learning of techniques, characterizing an excellent result.

## DISCUSSION

Laparoscopic surgery was a surgical advance and proved to be beneficial because the laparoscopic access reduces surgical trauma and shortens the surgery time as it does not require laparotomy and subsequent suturing of the abdominal wall. It also decreases postoperative pain, patient hospitalization time and prompt recovery of the patient with early return to their work and physical activities.

It is proven and validated that realistic simulation can be transferred to the operating room, saving the patient from learning. As a foresight of the future, it is evident that each teaching hospital and medical school could have a surgical skills laboratory in its methodological context of teaching, continuing education and research^8^.

The training assists the acquisition of skills for undergraduate, medical residency and graduate students. It is not justified to use patients on the so-called learning curve, until the surgeon acquires the skill, experience and proficiency. The initial skill must be acquired in experimental surgery laboratories and in simulators^8^.

In the theoretical-practical class, there is a lack of concern in the execution of tasks, with the possibility of making mistakes and repeating the training until they get it right, thus minimizing the psychological impact, stress, insecurity, anxiety about the risk of error and iatrogenics in a situation of hospital practice^8^.

With the emergence of laparoscopic surgery, the need and the challenge of training surgeons to acquire the necessary skills to practice efficiently and safely emerged^9^. Videosurgery introduced concepts that demand teaching models for the acquisition of skills, among them, the fulcrum effect with inverted movement, loss of tactile sensation and lack of depth perception, among other difficulties inherent to the technique. These factors decisively contribute to the learning curve of laparoscopic procedures^10^. Our work presented results that indicated a positive contribution to learning, meaning relevant findings to be considered in learning curves, mainly due to the fact that there are no prerequisites for learning a suture technique.

Farquharson et al.^11^ sought to build a reinforced feedback mechanism using the video assistance feature during the acquisition of surgical skills, compared to standard verbal feedback alone. In our work, the findings are in agreement as we also proved the reinforcement of learning feedback using graphic animation.

Works that related the gender of the participant with the final result of the course were found in the literature in other studies. In the research by Moura Junior et al.^8^ we analyzed a group of 49 students who were already attending and completed the basic courses of surgical technique and surgical clinic in the periods from 2010 to 2013, with a mean age of 24.7 years, male predominance, 33 students (67.35%) on females, 16 students (32.65%). In our study, the group of students was larger (n=56) and we are in agreement with the work by Moura Junior et al.^8^, we also observed a predominance of males (n=38) in relation to females (n=18). The mean age of the male group (34.57 years) was higher when compared to the female group (29.61 years). In both groups, male and female, 90% of the students were aged between 23 and 35.3 years.

We found in the work of Farquharson et al.^11^ two groups of students performing the same exercises for two days. On the second day, the video review group showed significant progress, with maximum scores, compared to the standard feedback group, in the specific domain of instruments, manual suturing with needles, accuracy in movements and in the execution of tasks, showing that the addition of Video can help provide feedback for surgical skills acquisition and can be incorporated into the formal surgical curriculum. In our work, we used the reinforcement of learning feedback using graphic animation, in agreement with Farquharson et al.^11^, where the addition of feedback with video or graphic animation favors learning.

Buckley et al.^12^ consistently proved the positive impact of simulation on operative time, although data are insufficient to demonstrate the transfer of these skills to the operating room.

Orzech et al.^13^ showed that training in virtual or conventional simulators significantly reduces the learning curve in laparoscopic sutures.

Choy & Okrainec^7^ showed that training in simulators improves residents’ ability to perform advanced procedures and the safety of residency programs.

Based on the evaluation of the students in our course, the importance of suture training in surgical training was observed, which is in agreement with the authors Buckley et al.^12^, Orzech et al.^13^ and Choy & Okrainec^7^.

The effectiveness of a concise two-day course of laparoscopic suturing was demonstrated by Palter et al.^14^. The study emphasized the fact that a certain minimal base of laparoscopic experience is not a prerequisite for learning a complex minimally invasive skill in a simulated environment.

In our study of a total of 56 students, although 57% did not present any training with endosuture, all of them showed satisfactory results, in agreement with the results indicated by Palter et al.^14^.

Regarding the evaluation of the course, other works with this analysis were found in their results. In Moura Junior et al.^8^ analyzing a group of 49 students, his course presented an excellent result in the evaluation of the students, featuring a percentage of 83.3% of positive answers for the six questions presented. Our results were similar, in agreement with the results of Moura Junior et al.^8^ with 100% approval for five of the six items: benefit from the course, improvement in thread manipulation, improvement in needle manipulation, improvement in suturing and indication of the course to another surgeon. In terms of operating safety, the acceptance percentage was 85.7%.

Our study had some limitations that make the simulation white box experience not entirely identical to the real surgical procedure. The lack of bleeding, which can occur during the surgical procedure, and the lack of camera tremor are situations that are not reproducible in a simulation course. The texture of silicone, although it resembles the tissue of human viscera, is in fact not identical. However, even with these limitations, our training contributed to the training and knowledge of surgical techniques for the physicians who participated in the course and with our findings, we can add that courses using black box are of great value in the training of the surgeon.

## CONCLUSION

Laparoscopic knot training, in silicone molds, in courses lasting 8h, proved to be effective.

Factors such as gender, age, laterality, prior knowledge in endosuture and medical specialty do not interfere in the learning results.
